# Long-term exposure to polypharmacy impairs cognitive functions in young adult female mice

**DOI:** 10.18632/aging.203132

**Published:** 2021-06-02

**Authors:** Eroli Francesca, Johnell Kristina, Latorre-Leal María, Hilmer Sarah, Wastesson Jonas, Cedazo-Minguez Angel, Silvia Maioli

**Affiliations:** 1 Karolinska Institutet, Department of Neurobiology, Care Sciences and Society, Center for Alzheimer Research, Division of Neurogeriatrics, Solna, Sweden; 2 Department of Medical Epidemiology and Biostatistics, Karolinska Institutet, Stockholm, Sweden; 3 Kolling Institute, Royal North Shore Hospital and University of Sydney, Sydney, Australia; 4 Aging Research Center, Karolinska Institutet and Stockholm University, Stockholm, Sweden

**Keywords:** polypharmacy, adverse outcomes, memory, female mice, sex-differences

## Abstract

The potential harmful effects of polypharmacy (concurrent use of 5 or more drugs) are difficult to investigate in an experimental design in humans. Moreover, there is a lack of knowledge on sex-specific differences on the outcomes of multiple-drug use. The present study aims to investigate the effects of an eight-week exposure to a regimen of five different medications (metoprolol, paracetamol, aspirin, simvastatin and citalopram) in young adult female mice. Polypharmacy-treated animals showed significant impairment in object recognition and fear associated contextual memory, together with a significant reduction of certain hippocampal proteins involved in pathways necessary for the consolidation of these types of memories, compared to animals with standard diet. The impairments in explorative behavior and spatial memory that we reported previously in young adult male mice administered the same polypharmacy regimen were not observed in females in the current study. Therefore, the same combination of medications induced different negative outcomes in young adult male and female mice, causing a significant deficit in non-spatial memory in female animals. Overall, this study strongly supports the importance of considering sex-specific differences in designing safer and targeted multiple-drug therapies.

## INTRODUCTION

Polypharmacy is defined as the concurrent use of five or more drugs [1] and is very common in older adults, who are the largest consumers of medications. The prevalence of polypharmacy has increased in many countries over the last decades [2] and the use of multiple drugs is becoming a burden on individuals and healthcare systems worldwide.

Most medications show good safety profiles when taken alone as monotherapies and correctly. However, the risk of developing adverse events can raise considerably when they are combined with other drug therapies, due for example to drug-drug interactions, prescribing cascade, and medication errors [3–5]. Furthermore, older adults are particularly vulnerable to negative effects of multiple-medication treatments due to both age-related physiological changes and more frequent occurrence of multiple pathological conditions (i.e., arthritis, cardiovascular diseases, osteoporosis, renal dysfunction, dyslipidemias) as a consequence of longer survival with chronic disorders. In the older population polypharmacy has been associated to a higher risk of several negative outcomes which comprise falls, hospitalization, higher frailty, and mortality [6–10].

Nevertheless, there is little experimental data about the potentially negative effects caused by polypharmacy and on the mechanisms behind these effects [11]. Drug safety studies often exclude older patients and are limited to monotherapies. Another important aspect which is poorly investigated is the influence of sex in drug use and response [12]. This is of particular importance in older adults since they often have altered pharmacokinetics, pharmacodynamics, efficacy, and toxicity [13, 14] which have shown to change between men and women. [15–18]. Therefore, sex represents a relevant factor to take into account when investigating adverse events related to polypharmacy.

We recently performed a study to explore the effects of long-term concomitant administration of five different medications on locomotion, anxiety, and cognition in mice [19]. The drugs included in the polypharmacy treatment were the most frequently used by older adults in Sweden [20], and among the most frequently used drug classes also in other European countries [21–24]. Importantly, we observed that polypharmacy impaired exploration and cognitive functions in young adult wild-type male mice [19].

In this study, female mice were administered the same polypharmacy regimen, containing aspirin, paracetamol, simvastatin, metoprolol and citalopram, with the aim of investigating the effects of multi medications in female animals and allow comparison with our previous study in young adult male mice [19]. Animals were fed with the polypharmacy diet and then assessed for locomotor function and coordination, cognitive tests, and anxiety-like behavior. Hippocampal tissues were analyzed to measure any changes in protein markers which could be related to the behavioral outcomes observed in polypharmacy mice. The following parameters were monitored as basic health indices: food/water intake, body weight (BW), serum creatinine and alanine aminotransferase (ALT) levels.

## RESULTS

### Treatment tolerance and health parameters

**Figure 1:**
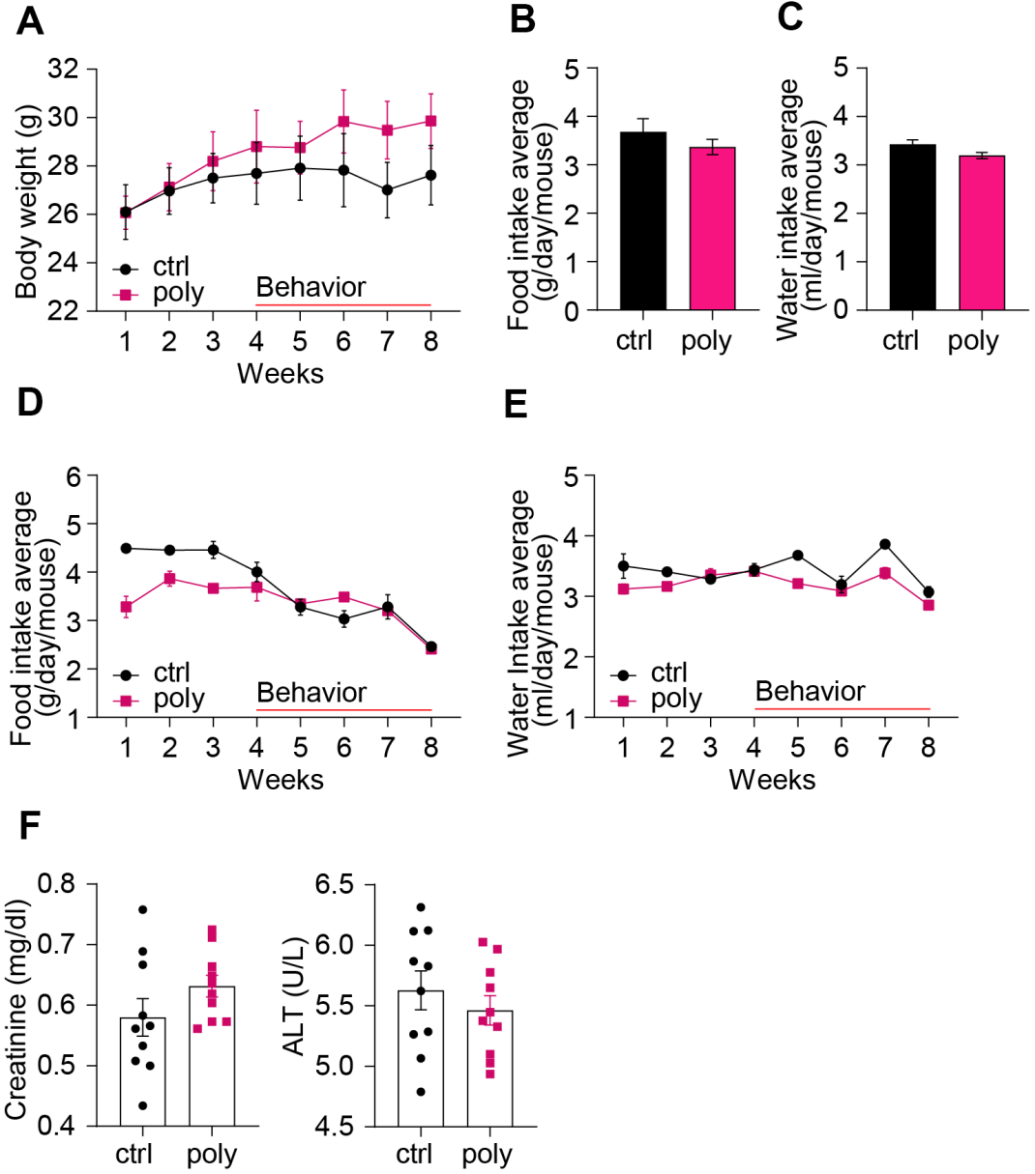
**Basic health parameters in control and polypharmacy treated mice: body weight, food and water intake, and serum proteins.** (**A**) The curves show mouse body weight measured weekly during the two months of control or polypharmacy diet. (**B**, **C**) The histograms represent the total average of food and water intake over the whole study period. (**D**, **E**) The curves show the weekly average of food and water intake monitored during the eight weeks of treatment. (**F**) Dot histograms express serum creatinine and ALT levels. Ctrl= control, Poly= polypharmacy, n= 10 animals per group. All data are presented as mean ± SEM.

The treatment was well tolerated by the animals and no increase in mortality was observed in polypharmacy fed mice compared to controls: all the mice reached the end of the study in good health. Polypharmacy fed mice showed a significant BW gain during the study period while controls did not (week 1 vs week 8: control group BW= 26 ± 1.2 g vs 28 ± 1.3 g, p= 0.09; polypharmacy group BW= 26 ± 0.7 g vs 30 ± 1.1 g, p= 0.001, two-way ANOVA repeated measurements; Figure 1A). No significant differences in mean food or water intake (FI, WI) were found between the two groups over the study period (Figure 1B, 1C), nor in the weekly average (Figure 1D, 1E). However, both controls and treated animals revealed a significant reduction of FI during the last 4 weeks (control group FI, week 3: 4.5 ± 0.2 g/day/mouse, week 8: 2.5 ± 0.1 g/day/mouse, p=0.02; polypharmacy group FI, week 3: 3.7 ± 0.1 g/day/mouse, week 8: vs 2.4 ± 0.1 g/day/mouse, p=0.02, two-way ANOVA repeated measurements; Figure 1D). The average FI was very close to the estimated one, therefore the drug concentrations taken by polypharmacy animals corresponded to the expected ones. Only in the last week the registered FI (2.4 ± 0.1 g, polypharmacy group, Figure 1D) was about 20% less than the anticipated one, meaning that the final drug dosage consumed was: 80 mg/Kg/day metoprolol, 80 mg/Kg/day paracetamol, 16 mg/Kg/day aspirin, 8 mg/Kg/day simvastatin and 8 mg/Kg/day citalopram, which is within the therapeutic dose range in humans for these medications [19].

As markers for renal and hepatic health status we measured serum levels of creatinine and ALT at the end of the treatment. Dot histograms in Figure 1F illustrate as there were no significant changes of the two markers between control and multiple-drug administered mice.

### Polypharmacy diet did not affect locomotor activity and anxiety-like behavior

**Figure 2:**
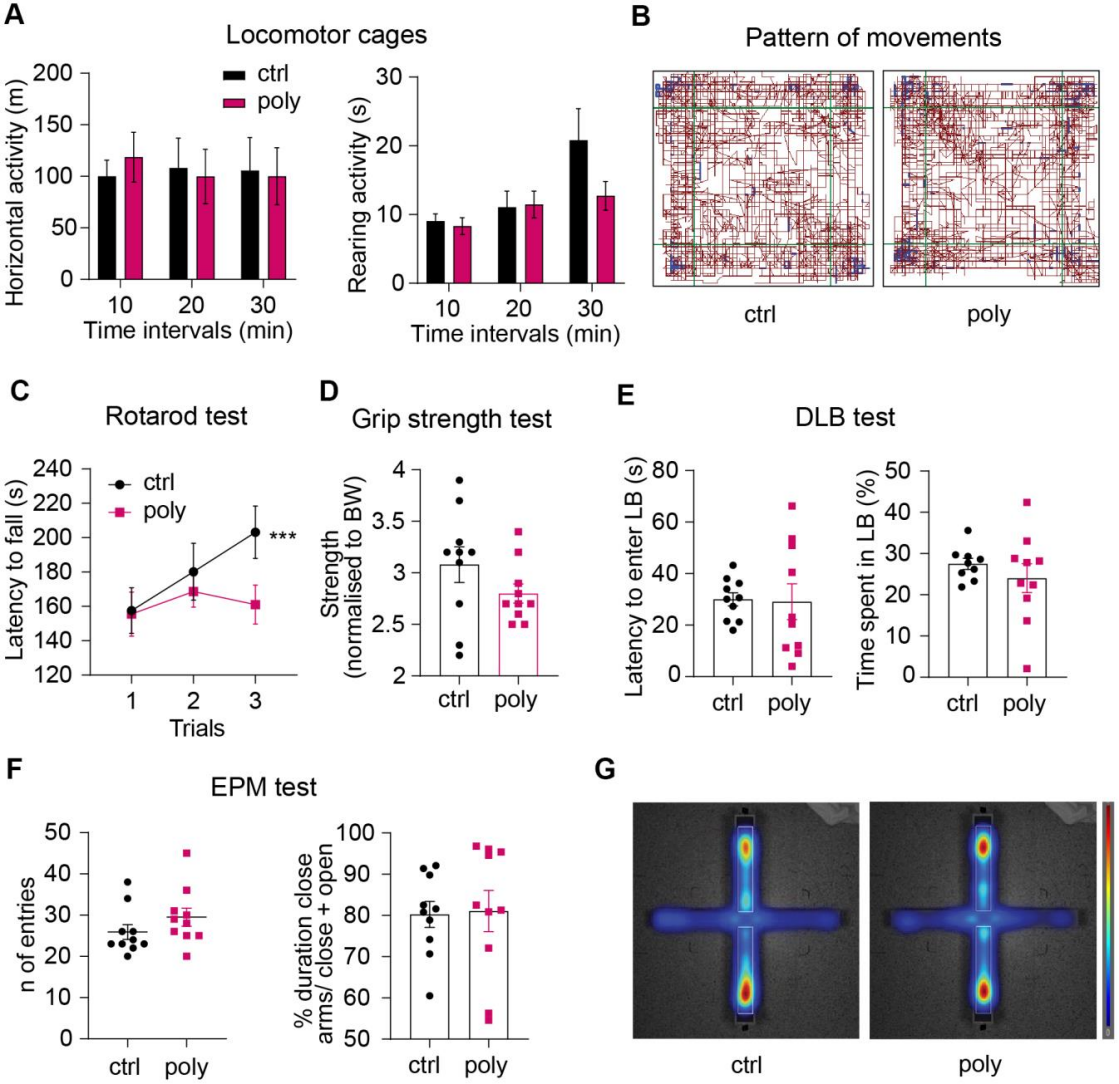
**Effect of polypharmacy regimen on locomotion, coordination and strength, and evaluation of anxiety-like behavior.** (**A**) Locomotor and explorative activity: histograms express horizontal and vertical activity (rearing) assessed in OF cages and analyzed per time intervals over a total duration of 30 minutes. (**B**) Representative map of the pattern of movements in a control and polypharmacy mouse during the 30-min trial in OF cages. (**C**) average of latency to fall measured over the 3-trial session of Rotarod test. Interaction between time and treatment groups were analyzed with two-way ANOVA repeated measurements; ***p≤0.001, trial 1 vs trial 3 in control group. (**D**) Dot histograms show the forelimb strength average measured by Grip Strength test in control and polypharmacy animals. (**E**) DLB test: dot plots express first latency to enter the LB and time spent by the mice moving in that area. (**F**) EPM test: number of entries and duration % of time spent in closed arms over the 5 min/trial. (**G**) Representative heatmaps of the EPM, where red zones display the area that the mice explored the most (average of control and polypharmacy group maps). Ctrl= control, Poly= polypharmacy, n= 10 animals per group. All data are presented as mean ± SEM.

We used open field (OF) locomotor cages to study general locomotor activity over a 30-min free exploration trial. Horizontal and vertical activity were analyzed over the total test duration and in time intervals of 10 min in order to monitor the habituation phase and the next exploratory patterns. The treatment did not alter the horizontal or rearing activity analyzed per time interval (Figure 2A), nor the total locomotion (horizontal activity: 241.6 ± 24 m vs 182.7 ± 13 m; rearing: 40.9 ± 6 s vs 32.4 ± 13 s, control vs polypharmacy group respectively, data not shown). The map in Figure 2B illustrates that control and polypharmacy animals showed a similar pattern of movements in locomotor cages.

Motor coordination and forelimb strength were assessed through Rotarod and Grip strength tasks. The analysis of latency to fall over the three Rotarod test trials showed that control mice significantly improved the performance on the rotor in trial 3 compared to trial 1 while polypharmacy mice did not (Figure 2C). Despite this there were no significant differences between the two groups. The outcomes from Grip strength test did not highlight relevant differences between control and treated animals in the front limbs force measured during the grid pulling (Figure 2D).

To explore whether the multiple-drug regimen could affect anxiety-like behavior we performed Dark/Light Box (DLB) and Elevated Plus Maze (EPM) tests. The results from DLB experiment showed that polypharmacy mice displayed a similar time spent in, and latency to enter, the lit compartment to the controls (Figure 2E). Likewise, EPM task did not reveal significant differences in the time spent by the animals exploring the close arms of the maze (about 80 % of the total trial duration: dot histogram in Figure 2F and heatmaps in Figure 2G).

### Polypharmacy regimen impaired object recognition and fear associated contextual memory

**Figure 3:**
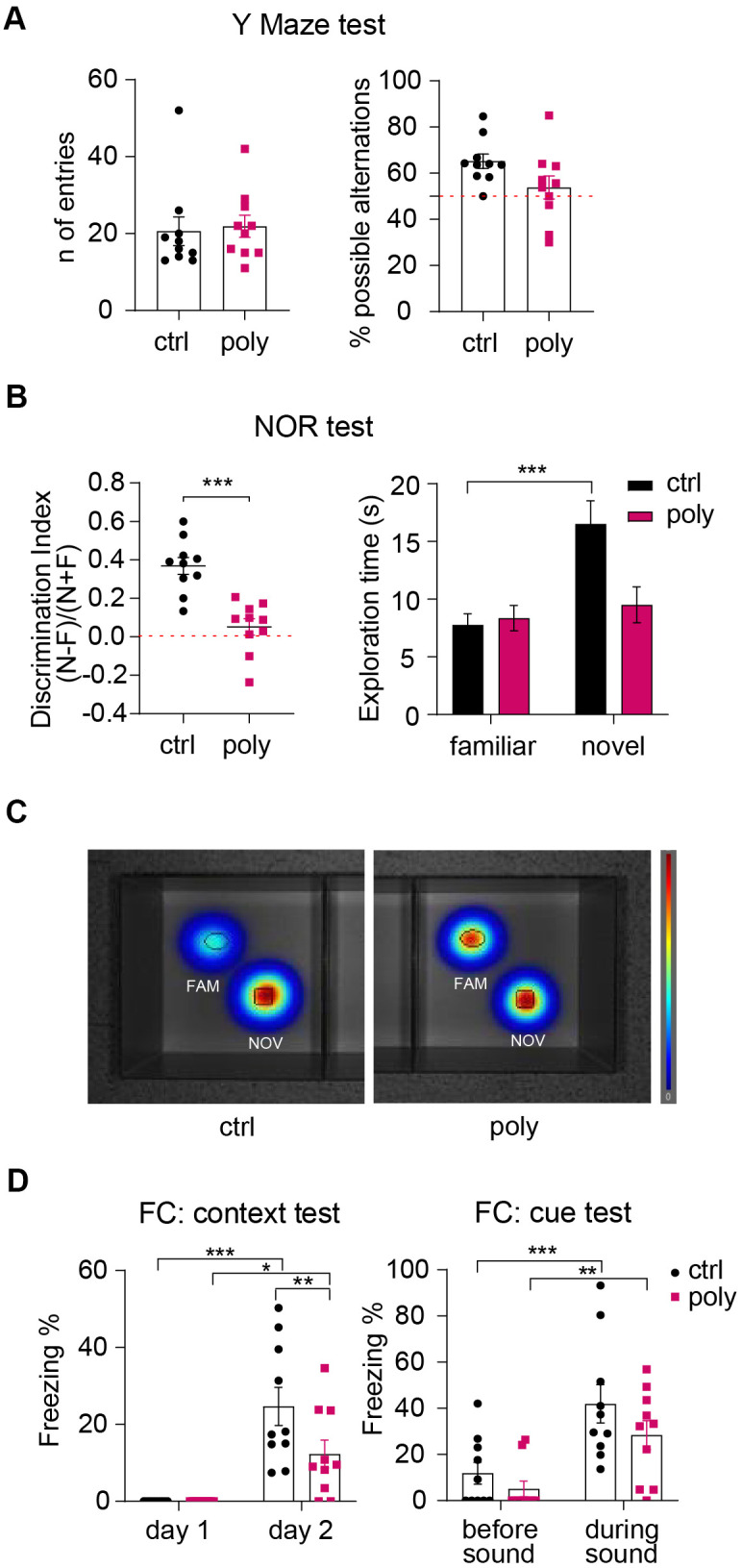
**Outcomes of cognitive tests: polypharmacy treatment impaired non-spatial memory.** (**A**) Number of entries and percentage of spontaneous alternations performed by control and polypharmacy mice in the Y Maze test. (**B**) NOR test, day 3: the dot plots express the discrimination index (a score above 0 indicates that the mice explored the novel object more than the familiar one). *p<0.001, t-Student test. Histograms on the right show the average of time spent in exploring the two objects by control and polypharmacy animals, ***p<0.001, two-way ANOVA repeated measurements. Note that control mice spent about double the time exploring the new object compared to the familiar one; on the contrary, treated animals did not differentiate between the two objects, as indicated by the discrimination index. (**C**) The heatmaps visually represent day 3 of NOR test and specifically the area explored around the objects by the animals, showing that only in control group there is a clear preference for the novel object compared to the familiar (in red color the most visited areas). Fam and Nov = familiar and novel object respectively. (**D**) Contextual and cue FC test: the graph on the left shows the percentage of freezing time measured on day 1 (habituation phase) vs day 2 (context test); the graph on the right expresses the freezing percentage measured before vs during the cue stimulus (sound). *p<0.05, **p<0.01, ***p<0.001, two-way ANOVA repeated measurements test. Ctrl= control, Poly= polypharmacy, n= 10 animals per group. All data are presented as mean ± SEM.

Mice underwent cognitive tasks to investigate the effect of the polypharmacy treatment on different types of memory and learning. To study spatial working memory, we ran the Y Maze test. Animals from both the groups performed a similar number of arm entries and a percentage of possible alternations above 50 on average (Figure 3A), suggesting that polypharmacy regimen in young adult female mice does not affect spatial working memory.

Non-spatial memory was investigated via Novel Object Recognition (NOR) test. On day 3, control mice exhibited a clear preference in exploring the novel object compared to the familiar one. Conversely, polypharmacy animals did not discriminate between the familiar and the novel object as they spent a similar time exploring both (Figure 3B, right panel). This was confirmed by the calculation of the discrimination index which was significantly higher for controls compared to treated mice, that presented an index close to 0 on average (Figure 3B, left dot plot). Heatmaps in Figure 3C represent by colors as control animals spent more time on the novel object (in red) while the polypharmacy mice stayed similarly on both. The outcomes from NOR test propose that multiple-medication regimen impaired non-spatial object recognition memory.

Fear conditioning (FC) test was performed to assess fear associated memory and learning. Mice were subjected to an auditive stimulus (cue) paired to a foot shock on day 1 and then tested for context and cue memory on day 2 and 3 respectively. The freezing % recorded during the habituation phase of day 1 (as a measure of baseline freezing) was compared to freezing % of day 2 to evaluate the context memory. To assess the cue memory, we measured the freezing % on day 3 before and during the sound stimulus. During the context test on day 2, both controls and treated animals showed a significantly increased freezing behavior compared to day 1 (Figure 3D, left graph). However, control mice responded to a greater extent to context recognition showing a significant higher freezing % than the polypharmacy ones (Figure 3D, left plot: **p=0.01, two-way ANOVA repeated measurements), indicating that the multi-medication treatment may affect FC contextual memory in young adult female mice. On day 3, we measured freezing % before and during the delivery of the acoustic stimulus; mice from control and polypharmacy administered group expressed a significantly stronger freezing behavior during cue application compared to before (Figure 3D, right graph), suggesting that both groups were able to associate the auditory cue to the adverse stimulus (the foot shock).

### Polypharmacy reduced the levels of memory-related proteins in hippocampus

**Figure 4:**
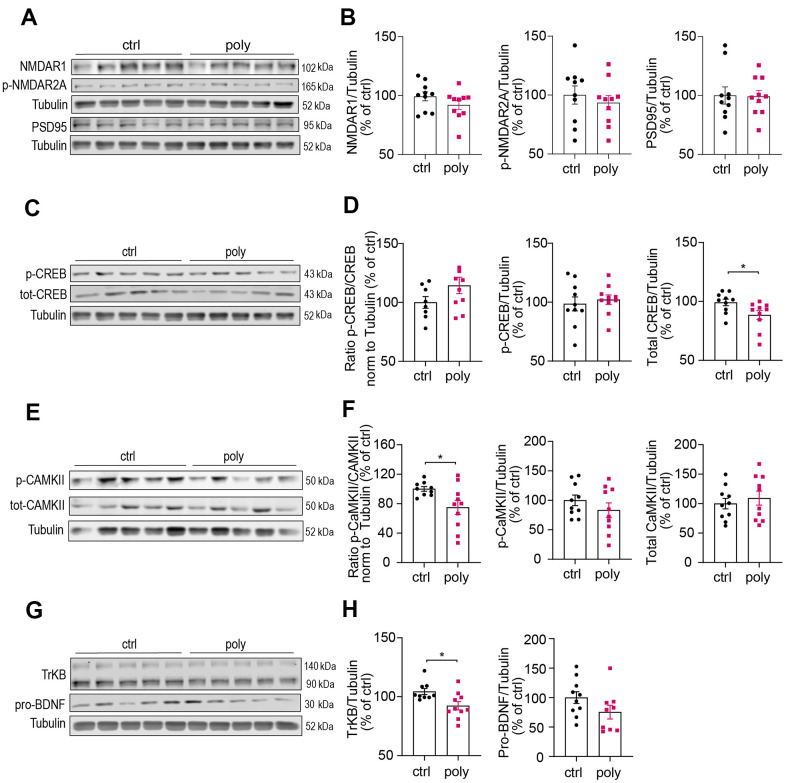
**Immunoblotting analysis of hippocampal protein levels in control and polypharmacy fed mice.** Hippocampal tissue lysates of control and polypharmacy mice were analyzed by western blotting experiments. Representative immunoblots and quantification of: NMDAR1, phospho-NMDAR2A and PSD95 proteins (**A**, **B**), phospho- and total-CREB, and ratio (**C**, **D**), phospho- and total-CAMKII, and ratio (**E**, **F**), TrkB and pro-BDNF proteins (**G**, **H**). *p<0.05, Mann-Whitney (**D**, **H**) or t-Student test (**F**). Total protein levels were normalized with respect to α tubulin. Ctrl= control, Poly= polypharmacy, n= 10 animals per group. All data are presented as mean ± SEM.

Western blotting experiments were performed to investigate whether the treatment could lead to changes in levels of hippocampal proteins involved in regulating synaptic plasticity and memory formation. We first analyzed the expression of synaptic N-Methyl-D- aspartate (NMDA) receptors (subunits NMDAR1 and phospho-NMDAR2A) and postsynaptic density protein 95 (PSD95), that are known to play a key role in synaptic transmission and potentiation and were found to be downregulated in our previous study on male mice [19]. Interestingly, we did not observe changes in the hippocampal levels of these markers between control and polypharmacy animals, as illustrated by immunoblots and histograms in Figure 4A, 4B. Since the data from NOR test indicated a clear memory impairment in treated mice, we explored markers for specific signaling pathways implicated in recognition memory. In the hippocampi of multiple-medication fed animals we found a decrease of total cAMP Response Element-Binding Protein (CREB) levels compared to controls, although the ratio of phospho/total CREB remained unchanged between the two groups (Figure 4C, 4D). The analysis of Ca^2+^/calmodulin-dependent protein kinase II (CaMKII) revealed a significant reduction of phosphorylated CaMKII in polypharmacy mice, as shown by the ratio of phospho/total protein (Figure 4E, 4F). The brain-derived neurotrophic factor (BDNF)–tyrosine kinase B (TrkB) signaling is another important system that regulates synaptic plasticity and is involved in recognition memory consolidation [25] and fear conditioning learning [26]. We quantified the levels of TrkB and pro-BDNF and we observed a significant downregulation of TrkB receptor expression in treated animals compared to controls (Figure 4G, 4H, left histogram). The levels of hippocampal pro-BDNF in polypharmacy mice resulted in a reduction of 25% on average than control levels (right plot of Figure 4H), albeit not significant (p=0.12, t-Student test).

## DISCUSSION

In this study we performed a preclinical investigation on the adverse events related to polypharmacy on locomotion, anxiety, and cognition in female animals. Previous studies reporting negative outcomes associated with multiple-drug use on animal models, including our recent study [19], were performed primarily in males [27, 28], except for one recently published study on physical functions in C57BL/6 male and female mice [29]. In the elderly population, women are more frequently exposed to polypharmacy and observational studies have reported a higher risk of receiving potentially inappropriate prescriptions in women compared to men [12, 30, 31].

In the current study we found that polypharmacy treatment significantly impaired object recognition and affected fear associated contextual memory, together with a significant decrease of some hippocampal proteins involved in pathways regulating the formation and consolidation of these types of memories. Noteworthy, we did not observe the impairments in explorative behavior and spatial memory that we previously reported in young adult male mice administered the same polypharmacy diet. We believe that the results from this study give interesting insights about possible sex-specific adverse effects from multiple-drug use and support the need of more targeted multi-medication therapies which consider sex-related differences.

Animals were administered the polypharmacy regimen for eight weeks and tested for behavioral experiments during the last four weeks of treatment. The diet was well tolerated, serum levels of hepatic and renal function did not change between control and treated animals, nor we did observe signs of illness among the mice. The decrease in FI observed during the last four weeks in both the control and treated group correlates with the behavioral assessment period which may induce stress in mice as we have previously observed [19]. The average FI was similar between the two groups over the study period, except for a lower FI baseline during the first week in the polypharmacy animals compared to controls. Despite this, we observed a significant increase of BW in the polypharmacy fed mice compared to controls. This BW gain might be due to a metabolic effect caused by one or more specific drugs contained in the polypharmacy diet. Mild weight gain can manifest as a side effect of some beta blockers, including metoprolol [32], and antidepressants like citalopram [28, 33]. Also, it might be due to the presence of simvastatin in the drug combination: use of statins in adults has been associated with an increase in body mass index in comparison with statin nonusers [34]. Interestingly, this increase in BW was not reported in male mice administered with the same multi-medication therapy [19]. In this regard it is relevant to note that side events for statin use, like muscle pain, have been found to affect women with a higher prevalence compared to men, together with a lower efficacy of the lipid lowering action in women than in men [35, 36]. This supports the fact that drug outcomes may vary with sex.

When behavior was assessed in mice, no differences between treatment groups were found in the locomotor and exploratory patterns recorded in OF cages, indicating that polypharmacy administration in adult female mice did not affect exploration and total locomotor activity. This finding differs from our data in young adult male mice [19] indicating that female mice could be more resilient to these effects than males treated with the same polypharmacy combination at young adult age. Huizer-Pajkos et al. reported that a shorter treatment of 4 weeks in young male mice did not lead to impairments in OF [27], while data from Mach et al. report reduction of distance traveled in OF after 12 weeks of polypharmacy treatment in middle aged male mice, as well as after 12 months of low drug burden index and high drug burden index polypharmacy treatments in aging male mice [28]. Interestingly, functional outcomes for motor coordination and balance in Rotarod did not differ between control and polypharmacy female mice. However, while control group improved significantly during the 3 trials of Rotarod, polypharmacy mice showed no improvement and unchanged latencies to fall among trials, suggesting that multi-medications in female mice could start to affect coordination and balance at young adult age. Previous studies on aging male mice reported that performance in Rotarod test was negatively affected by the polypharmacy treatment [27]. Moreover, the observed lack of improvement during the Rotarod task may be also caused by decreased motor learning in the polypharmacy group rather than coordination deficit only [37]. OF and Rotarod tests resulted in different outcomes in adult males and females treated with our selected drug combination, supporting possible sex-specific adverse effects in locomotor functions of multi-medication therapies. A recent study in young and old male and female C57BL/6 mice found no significant difference between young males and females in baseline grip strength, motor coordination, gait speed, distance travelled in the open field, anxiety or nesting [29], suggesting that the sex-specific outcomes we observe here are not related to baseline differences in the behavioral performance between sexes.

Our previous study was the first to investigate the effects of polypharmacy on cognitive functions in mice and we reported that a combination of different medications had a negative effect on spatial working memory in Y Maze and reduced hippocampal postsynaptic proteins already at young age [19]. Interestingly, when the Y Maze test was performed in female mice no differences were found between groups. These results were further confirmed by western blot analyses of proteins mainly involved in formation and consolidation of spatial memory as NMDA receptors and PSD95: female mice administered with polypharmacy did not show a reduction of these markers in hippocampus when compared to controls. It is important to mention that NMDAR1 and NMDAR2A/B expression were reported to be higher in the hippocampus and in postsynaptic density fractions of adult female mice than in those of males [38]. This aspect may influence the sex-specific effect of polypharmacy on postsynaptic protein levels observed in our studies.

Noteworthy, the present study shows that multi-medication therapy in female mice impaired object recognition memory, measured by the ability to remember an object previously encountered and therefore distinguish a novel object from a familiar one in the NOR test. This type of memory was not affected by the same treatment in male mice [19]. Several studies reported that a functional hippocampus is essential for the formation of recognition memory in rodents [39, 40]. Within hippocampus, several signaling cascades have been shown to be critical for consolidation of this type of memory. Specifically, CREB inactivation in CA1 and CaMKII inactivation in mutant mice has been shown to impair long term object recognition memory [41, 42]. In the hippocampi of polypharmacy female mice, a decrease in total levels of CREB as well as a decrease in phosphorylation of p-CaMKII was shown. These results are consistent with the behavioral findings in the NOR test.

In addition to Y Maze and NOR we performed FC in order to assess fear associated memory. In this test, control and polypharmacy mice learned to associate both the context and the cue to the adverse event of the foot shock. However, it must be pointed out that during the context test polypharmacy mice showed a significant lower freezing time than control animals. This result shows that polypharmacy mice performed worse when associating the foot shock to the context, suggesting that the multi-medication treatment affected the consolidation of fear associated contextual memory in female mice at young age. The FC deficits observed in treated mice are not as consistent as in the NOR test and we may hypothesize that aging would further lead to greater deficits in fear associated memories caused by the current combination of multiple drugs in female mice. This hypothesis is supported by western blot analyses revealing a significant reduction of TrkB levels in hippocampus of treated females as compared to controls. BDNF–TrkB pathway is a ligand–receptor system that underlies synaptic plasticity and has been shown necessary for acquisition and consolidation of fear conditioning in different brain regions including hippocampus [43–46]. While we observed a decrease in TrkB receptors, we did not observe a significant reduction in BDNF levels in hippocampus of treated mice, and it is possible that a longer multi-medication treatment as well as aging would eventually lead to a greater reduction of BDNF in female mice, followed by a greater deficit in FC test. Additionally, phosphorylation of CREB mediated by CaMKII may as well affect BDNF levels [47]. In this context, it is important to mention that a large body of evidence reported that CaMKII-CREB signaling is participating in estrogen receptor signaling in the brain [48, 49]. Brain estrogen signaling has a neuroprotective role and is essential for synaptic function. The multi-medication therapy proposed in this study could affect estrogen signaling, further supporting the sex-differences in outcomes as different types of memories.

To our knowledge, the use of the individual drugs composing our polypharmacy regimen has not been reported to induce toxic effects in mice [27, 50–54]. This suggests that the combination of different medications used in this study causes the negative outcomes observed. However, only one out of the six pre-clinical studies cited above has been conducted in both male and female mice, while the rest used only male animals. This may lead to a more difficult interpretation of the data on the effects of polypharmacy in female mice. For instance, previous research on monotherapies in rodents did report different results within male and female animals: a recent study on treatments for post-traumatic stress disorder found differential effects caused by citalopram on fear associated memory in female mice compared to males [55]; similarly, different outcomes were found after administration of metoprolol: this beta-blocker impaired performance in Morris Water Maze and FC tests in males of the APP Alzheimer's Disease mouse model and wild-types but not in females [56]. Aspirin was reported to increase the lifespan of male mice but not of females [57], and this was attributed to different drug metabolism and disposition between sexes. These observations support the idea that more targeted research is necessary to refine appropriate therapies taking into account sex-specificity.

There are some limitations to consider in this study. Female and male experiments were not conducted simultaneously, not allowing a statistical comparison between male and female groups. While we replicated laboratory conditions, there may have been experimental differences that affected the behavioral outcomes. The study has been conducted at young adult age. Although there is some evidence of multiple-drug use in young and adult subjects [58], and its prevalence over time have been increasing in younger age groups [59] polypharmacy is more frequent in old age. Therefore, the use of aged mice would be of great interest to discuss the effects of polypharmacy related to older population. To do so, an optimization of the experimental design will be necessary for future studies in old animals. A recent study on the effects of a different polypharmacy regimen on physical function in young and old male and female mice has been published, demonstrating a marked increase in susceptibility to functional impairment in old age and greater impact on grip strength in males than in females [29]. The investigation of the impact of age and sex on susceptibility to the effects of polypharmacy on cognitive function can be the subject of future studies.

Taken together, this study is relevant and highlights the importance of investigating the possible adverse effects of multiple-medication treatments in female mouse models in the future. This is one of the first reports of the effects of polypharmacy in female mice and the first to study its cognitive effects. The fact that polypharmacy induces strong impairments in different types of memory and decreases synaptic proteins already at young age is significant and support the importance to further explore adverse effects of the multiple-drug regimen in old mice. The results from this study will therefore be useful to design and interpret future results on aging animals. The same combination of medications including simvastatin, metoprolol, aspirin, paracetamol, and citalopram induced clearly distinguished effects in male and female young adult mice, that can be translated to humans. In sum, this study strongly supports the importance of considering sex-specific differences in designing safer and targeted multiple-drug therapies for older adults.

## MATERIALS AND METHODS

### Animals

In this study we used wild-type C57BL/6J female mice, which were purchased from Janvier Labs (France) at the age of 8 weeks and then housed in our animal facility in groups of five per cage (Karolinska Institutet, Solna, Sweden) with 12-h light/dark cycle, ad libitum access to food/water and standard enrichment (cardboard tunnels, wooden sticks, and tissue paper). A control and a polypharmacy group of 10 animals each were randomly constituted in groups of 5 mice per cage, when the mice were 5.5 months old. We used a standard rodent diet (control diet) to feed the control group: 18.5 % proteins, 5.5 % oils and fats, 4.5 % fiber (Teklad 2918 diet, Research Diet Inc., NJ, USA) while the polypharmacy group was administered with the same diet supplemented with drugs (polypharmacy diet).

### Polypharmacy treatment and study plan

The drugs for the polypharmacy regimen were chosen based on the most frequently used medications in older population in Sweden [20]: metoprolol (100 mg/Kg/day; Sigma-Aldrich, USA) [60], paracetamol (acetaminophen, 100 mg/Kg/day; Sigma-Aldrich, USA) [61], aspirin(acetylsalicylic acid, 20 mg/Kg/day; Sigma-Aldrich, USA) [54], simvastatin (10 mg/Kg/day; Selleck Chemicals, USA) [62] and citalopram (10 mg/Kg/day; Selleck Chemicals, USA) [63]. Paracetamol was selected as analgesic as it is the second most frequently prescribed drug to older adults with polypharmacy in Sweden [20]. Many older adults have chronic pain and paracetamol is considered first line treatment for acute and chronic pain in older people, having a more favorable safety profile than non-steroidal anti-inflammatory drugs (NSAIDs) and opioids [64]. Aspirin was included in the regiment for its antiplatelet properties, which is used for prevention of cardiovascular and cerebrovascular disease and low dose aspirin is among the three most commonly used drugs in older adults in Sweden [20].

Compound dosages per Kg/BW were selected after translation from the human therapeutic range into the mouse one and according to previous studies where they did not show toxicity in rodents, as explained in detail in our polypharmacy pilot study in young wild-type male mice [19]. Taking into account some variability between the estimated FI and the real one we decided to keep the drug concentrations towards the higher therapeutic dose, with the exception of drugs with potential dose-dependent toxicity in rodents (i.e. paracetamol [65, 66]). Medicine concentrations per Kg/diet were considered based on a FI on average of 0.1 ± 0.2 g food/g mouse/day as previously observed for C57BL/6J mouse strain in our animal facility and literature [19, 67].

According to our pilot study design [19] the animals were assessed for behavioral studies after four weeks of treatment, at 6.5 months of age, while carrying on the polypharmacy regimen for other four weeks, for a total duration of eight weeks. Over the study period we monitored the following parameters weekly: BW, FI (g food/mouse/day) and WI (ml water/mouse/day). Every week the chow was replaced with fresh food. At the end of the two-months treatment period the animals were sacrificed by cervical dislocation and trunk blood was collected. After brain dissection, tissues were collected and immediately snap frozen in dry ice and stored at -80 C until further use.

### Ethical statement

All behavioral experiments were run in accordance with the local national animal care and guidelines and approved by the local committee of Karolinska Institutet and the Swedish Board of Agriculture (ethical permit ID 827). All possible efforts were made to reduce any suffering or distress to the animals.

### Behavioral tests

Mice were evaluated with the following behavioral tests after four weeks of treatment at 6.5 months of age: Open Field (OF), Rotarod, Grip Strength, Elevated Plus Maze (EPM), Dark/Light Box (DLB), Y Maze, Novel Object Recognition (NOR) and Fear Conditioning (FC). All the experiments were run between 9:00 and 14:00 by a female researcher (FE) with a break from one to six days after those tests considered more stressful or physically demanding to allow the animals to recover. The order of the tasks was chosen according to the level of stress caused by the protocol, starting from the least stressful test: OF, EPM, DLB, Y Maze, NOR, Grip strength, Rotarod, FC [68]. Mice were allowed to acclimatize to the experimental room for 45 minutes prior to starting each test. The experiments were performed in white light. All the apparatuses were cleaned with 70% ethanol solution before starting each test and between animals.

OF activity in locomotor cages, Rotarod, EPM, DLB, Y Maze and NOR test protocols were run as recently described in detail [19]. Data of EPM, Y Maze and NOR experiments were acquired with a camera installed above the apparatus/boxes, connected to the video-tracking software Ethovision XT 15 (Noldus Information Technology, The Netherlands). OF and DLB tests were performed using 45 x 45 cm activity cages where the animal movements were automatically detected as infrared beam interruptions by TSE ActiMot software (TSE Systems GmbH, Germany). Horizontal and vertical activity in OF, as well as the latency and time spent in the light compartment in DLB tests were analyzed through the same software.

### Grip strength test

This test was used to evaluate the forelimb grip strength of the animals. The apparatus consisted of a grid attached to a force transducer which measured the force (in grams) applied by the mouse pulling the grid (Bioseb Instruments) [69, 70]. During the pull the mouse was held by the tail by the experimenter and only pulls using both forepaws were considered. The animals performed three series of 3-pulls each with a short resting period between each (2 minutes). The final grip strength was calculated by taking the average of the 9 measurements collected over the 3-pull series normalized for the BW.

### Contextual and cue FC test

This experiment was performed in transparent wall chambers with a stainless-steel grid floor which were enclosed in a soundproof apparatus (TSE Multi Conditioning Systems- TSE Systems GmbH, Germany). On day 1, mice were allowed to freely explore the context (a 20 x 20 x 40 cm square base chamber) for 2 minutes (habituation phase) and subsequently were exposed to a conditioned stimulus (55 dB sound at 5000 Hz, 30 sec duration) followed by a mild foot shock (0.3 mA, 2 sec duration). The sound-shock pairing was repeated three times in total with a 50-sec interval between each one. On day 2 (after 24 h) mice were returned to the same chamber for a period of 3 minutes to assess contextual fear memory. No sound or shock were given in this session. On day 3, the context was altered to evaluate the animals for cue memory [71]: the squared chamber was replaced with a round one (20 cm diameter x 40 cm high) and the grid floor was covered by a black smooth surface. To modify the odor, we cleaned the chamber with hypochlorous water instead of 70% ethanol. The animals were placed in this “new” context and after 2-minutes of free exploration they received the sound stimulus (same as in day 1: 55 dB at 5000 Hz) for a further 2 minutes continuously. The Freezing behavior (defined as complete absence of mobility within the same area for a time > 2 seconds) was measured through TSE Multi Conditioning software. The freezing % recorded during the habituation phase of day 1 (as a measure of baseline freezing) was compared to freezing % of day 2 to evaluate the context memory. To assess the cue memory, we measured the freezing % on day 3 before and during the sound stimulus.

### Immunoblotting analysis

We performed western blot experiments on hippocampal tissue lysates and protein levels were quantified after separation by acrylamide gel electrophoresis (gradient 12-7.5 %) and transfer to a nitrocellulose membrane, as previously described [19, 72]. Membranes were incubated overnight at 4° C with the following primary antibodies: rabbit anti-phospho NMDA receptor 2A (1:250, Abcam), mouse anti- NMDA receptor 1 (1:2000, BD Bioscience), mouse anti- PSD95 (1:1000, Abcam), mouse anti-CREB (1:750, Cell Signaling) and rabbit anti-phospho CREB (1:1000, Cell Signaling), rabbit anti-CaMKII and rabbit anti-phospho CaMKII (1:1000, Cell Signaling), rabbit anti-TrkB (1:1000, Cell signaling), rabbit anti-BDNF (1:1000, Abcam) and mouse anti-alpha-tubulin (1:30000; Sigma-Aldrich, USA). Incubations with secondary antibodies were done for 2 hours at room temperature with anti-rabbit or anti-mouse immunoglobulin G (IgG) at 1:10000 dilutions (LI-COR Biosciences GmbH, Germany). Immunoreactivity was detected with LI-COR® Odyssey® system (LI-COR Biosciences, USA) by infrared fluorescence and quantified with ImageJ 1.48v software (NIH, MA, USA) by densitometry analysis of the immunoreactive bands.

### Blood analysis

Trunk blood was collected right after the animal sacrifice and allowed to clot for 30 min at room temperature, followed by *5000 g* centrifugation for 10 minutes at 4° C to collect the serum fraction [19, 73]. Serum creatinine and ALT levels were measured using the following assay kits respectively: DICT-500 (BioAssay Systems) and MAK052 (Sigma-Aldrich). Assays were performed according to manufacturer instructions.

### Statistical analysis

All data are displayed as mean ± standard error of the mean (SEM), with n indicating the number of animals. We used GraphPad Prism 9 software (San Diego, CA, USA) to perform the statistical analyses. T-Student or Mann-Whitney tests were used when comparing the average of two groups for parametric and non-parametric data respectively. Data distribution was evaluated with Shapiro-Wilk test. When two independent variables were present two-way ANOVA repeated measurements, followed by Tukey's multiple comparison test, was used to analyze the data. A P value ≤ 0.05 was considered as index of significance.
